# An Alternative Pipeline for Glioblastoma Therapeutics: A Systematic Review of Drug Repurposing in Glioblastoma

**DOI:** 10.3390/cancers13081953

**Published:** 2021-04-18

**Authors:** Seán B. Lyne, Bakhtiar Yamini

**Affiliations:** Department of Neurological Surgery, University of Chicago Medicine and Biological Sciences, Chicago, IL 60637, USA; sean.lyne@uchospitals.edu

**Keywords:** glioblastoma, GBM, therapeutics, chemosensitization, radiosensitization, repurposing, repositioning

## Abstract

**Simple Summary:**

Glioblastoma is a devastating malignancy that has continued to prove resistant to a variety of therapeutics. No new systemic therapy has been approved for use against glioblastoma in almost two decades. This observation is particularly disturbing given the amount of money invested in identifying novel therapies for this disease. A relatively rapid and economical pipeline for identification of novel agents is drug repurposing. Here, a comprehensive review detailing the state of drug repurposing in glioblastoma is provided. We reveal details on studies that have examined agents in vitro, in animal models and in patients. While most agents have not progressed beyond the initial stages, several drugs, from a variety of classes, have demonstrated promising results in early phase clinical trials.

**Abstract:**

The treatment of glioblastoma (GBM) remains a significant challenge, with outcome for most pa-tients remaining poor. Although novel therapies have been developed, several obstacles restrict the incentive of drug developers to continue these efforts including the exorbitant cost, high failure rate and relatively small patient population. Repositioning drugs that have well-characterized mechanistic and safety profiles is an attractive alternative for drug development in GBM. In ad-dition, the relative ease with which repurposed agents can be transitioned to the clinic further supports their potential for examination in patients. Here, a systematic analysis of the literature and clinical trials provides a comprehensive review of primary articles and unpublished trials that use repurposed drugs for the treatment of GBM. The findings demonstrate that numerous drug classes that have a range of initial indications have efficacy against preclinical GBM models and that certain agents have shown significant potential for clinical benefit. With examination in randomized, placebo-controlled trials and the targeting of particular GBM subgroups, it is pos-sible that repurposing can be a cost-effective approach to identify agents for use in multimodal anti-GBM strategies.

## 1. Introduction

Glioblastoma (GBM) is the most common primary malignant brain tumor worldwide and is also the most lethal, with a median survival of approximately 15 months after diagnosis [1]. According to the recent analyses by The Central Brain Tumor Registry of the United States (CBTRUS), GBM comprises 48.3% of primary malignant brain tumors and has an overall five-year survival rate of 6.8% [2]. Currently, treatment for GBM involves maximal surgical resection followed by combination radiation therapy (RT) and chemotherapeutics, most often involving the alkylating agent, temozolomide (TMZ). In addition, the adjunctive use of tumor-treating field (TTF) technology has shown some survival benefit [3]. Despite this multimodal approach, overall survival (OS) and quality of life remain poor for patients with GBM [4,5].

While traditional drug development pipelines are hampered by high cost and low success rate, an alternative strategy is repurposing. Specifically, well-established drugs that were previously developed to treat other diseases can be used as potential antineoplastic agents. Repurposed drugs are particularly attractive as they already have well-established safety profiles and often require smaller cohorts for therapeutic efficacy analysis [6]. These factors help to streamline the passage of repurposed agents through the stringent FDA requirements for approval of novel agents. Repurposed drugs not only have a quicker approval time for an additional indication, but also have over 50% cost reduction in comparison to novel drug development [6], observations that are exemplified by the success of prior spontaneous and planned repurposing discoveries [7].

Important obstacles to repurposing drugs for GBM treatment include the presence of the blood–brain barrier (BBB), adverse effects when combining repurposed agents with other medications, intellectual property concerns and drug availability. The BBB can be bypassed using adjunctive strategies such as increasing its permeability or direct tumor cell targeting with nanocarriers. Notably, overcoming these obstacles can potentially affect the cost of production and delivery [8,9,10]. In addition, given the diverse molecular nature of GBM, it is important to appreciate that novel therapeutics may only be beneficial for specific subsets of patients. While DNA damage repair factors such as methylguanine methyltransferase (MGMT) or mismatch repair (MMR) activity are well known for modulating response to therapy, more recent prognostic factors such as isocitrate dehydrogenase (IDH) status also play a pivotal role in overall outcome [11,12,13,14,15]. This molecular variability plays a central role in the success of repurposed agents. In this regard, GBM can be clustered into several expression-based subgroups that not only have different genetic make up but also response to therapy [12,14,15]. These observations highlight the importance of examining novel repurposed agents in a variety of GBM subgroups to determine their overall potential for clinical use.

Identification of repurposing candidates has involved computational bioinformatics, large-scale screening experiments or candidate-based approaches [16,17,18]. Moreover, the examination of agents has ranged from cell-based studies to large multi-institutional randomized clinical trials. Here, we present a systematic review of repurposed agents used for the management of GBM. We categorize the findings into two groups based on their stage of development: those in the early stage (i.e., in vitro or animal model testing) and those that have transitioned to a clinical trial.

## 2. Materials and Methods

Preferred Reporting Items for Systematic Reviews and Meta-Analyses (PRISMA) guidelines were used for this study. A literature search was conducted using the PubMed database on 17 August 2020, with results restricted to articles written in the English language. The original search terms included glioblastoma, glioma, GBM, astrocytoma, drug, therapy, treatment, chemotherapy, radiotherapy, pharmacology, repurposing, repurposed, repurpose, repositioning, reposition and re-tasking. Any articles that were not fully available online were excluded from systematic review. Studies were selected for inclusion in the systematic review and analysis if they: (a) used a repurposed agent against GBM where the specific drug had been initially used to treat another disease; (b) used a repurposed agent as an adjunctive therapeutic in the surgical resection of GBM; or (c) used the repurposed agent for chemo/radiosensitization in GBM. No prespecified inclusion criteria regarding length of follow up, type of study or date of publication, were used. Capability of penetrating the BBB was not used as an exclusion criterion.

ClinicalTrials.gov was examined on 17 August 2020 as a second database to capture any clinical trials that did not have published results. Initial search criteria included glioma studies that were: (a) currently active and no longer recruiting patients; (b) currently recruiting; or (c) enrolling by invitation. Search criteria were limited to: pharmacologic interventional studies. An additional search was conducted with simple criteria including glioma and repurposed. The identified trials were then excluded or included on the basis of whether or not they were specifically interventional studies using a drug primarily developed for another disease.

The PubMed search yielded a total of 135 articles for screening. In accordance with eligibility for inclusion in this review, six previous review studies with no new novel meta-analysis results or data were excluded along with 14 articles that were outside the scope of this review. These latter articles fell under the following subheadings: (a) repurposing therapy for a disease other than glioma; (b) examination of a novel therapy for glioma; (c) studies that lacked an intervention; and (d) studies that used a therapeutic other than a chemical agent (e.g., RNA, virus or genetic manipulation). Based on the above criteria, a total of 115 articles remained for review. Any comments on original articles were examined and if the original article was not enveloped within the search, it was added. Relevant pivotal articles that were cited in an article identified in the literature search were included if they were not already included in the 115 articles. In addition, PubMed searches to encompass therapies targeting the tumor microenvironment (TME), major signaling pathways active in GBM and GBM stem cells, were performed, yielding a total of 15 novel articles that were not covered by the initial PubMed search.

**Figure 1:**
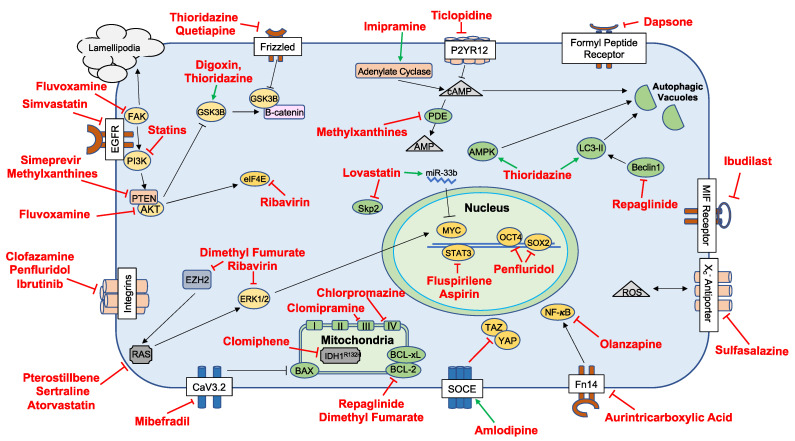
Cellular pathways and interactions detailing the key proposed mechanisms of drugs that have completed preclinical investigation and have yet to be fully studied in the clinical setting. Abbreviations: Adenosine diphosphate receptor (P2YR12); cyclic adenosine 3′,5′-cyclic monophosphate (cAMP); adenosine 3′,5′-cyclic monophosphate (AMP); phosphodiesterase (PDE); glycogen synthase kinase (GSK3B); phosphoinositide 3-kinase (PI3K); AMP-activated protein kinase (AMPK); microtubule-associated protein 1A/1B-light chain 3 phosphatidylethanolamine conjugate (LC3-II); signal transducer and activator of transcription 3 (STAT3); (sex determining region Y)-box 2 (SOX2); octamer-binding transcription factor 4 (OCT4); focal adhesion kinase (FAK); phosphatase and tensin homolog (PTEN); protein kinase B (AKT); eukaryotic translation initiation factor (eIF4e); S-phase kinase-associated protein 2 (Skp2); microRNA-33b (miRNA-33b); Macrophage migration inhibitory factor (MIF); reactive oxygen species (ROS); tafazzin (TAZ); yes associated protein (YAP); fibroblast growth factor-inducible 14 (Fn14); store-operated calcium entry (SOCE); isocitrate dehydrogenase 1 (IDH1); enhancer of zeste homolog 2 (EZH2); rat sarcoma (RAS); extracellular signal-regulated kinase (ERK); epidermal growth factor receptor (EGFR); t-type calcium channel (CaV3.2); Bcl-2-associated X protein (BAX); B-cell lymphoma 2 (BCL2); B-cell lymphoma-xL (BCL-XL); nuclear factor kappa-light-chain-enhancer of activated B cells (NF-KB).

**Figure 2:**
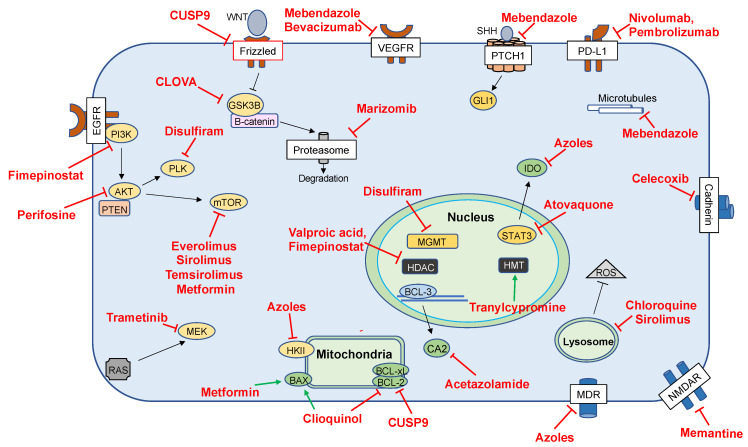
Cellular pathways and interactions detailing the key proposed mechanisms of drugs that have completed preclinical investigation and have also been studied in clinically. Abbreviations: Histone deacetylase (HDAC); O^6^-methylguanine-DNA methyltransferase (MGMT); histone methyltransferase (HMT); carbonic anhydrase 2 (CA2); muli-drug resistance protein (MDR); N-methyl-D-aspartate receptor (NMDAR); Indoleamine 2,3-dioxygenase (IDO); glioma-associated oncogene (GLI1); protein patched homolog 1 (PTCH1); programmed death-ligand 1 (PD-L1); polo-like kinase 1 (PLK); mitogen-activated protein kinase kinase (MEK); mechanistic target of rapamycin (mTOR); hexokinase II (HKII); glycogen synthase kinase (GSK3B); phosphoinositide 3-kinase (PI3K); signal transducer and activator of transcription 3 (STAT3); phosphatase and tensin homolog (PTEN); protein kinase B (AKT); reactive oxygen species (ROS); rat sarcoma (RAS); extracellular signal-regulated kinase (ERK); vascular endothelial growth factor receptor (VEGFR); endothelial growth factor receptor (EGFR); Bcl-2-associated X protein (BAX); B-cell lymphoma 2 (BCL2); B-cell lymphoma-xL (BCL-XL): B-cell lymphoma 3-encoded protein (BCL-3).

The ClinicalTrials.gov search conducted on 17 August 2020 yielded a total of 65 active clinical trials. These trials were either recruiting subjects or were active but no longer recruiting. Additionally, studies without results were included. Of the 65 active clinical trials, 35 met eligibility for inclusion in this review. Fourteen of the excluded trials investigated viral, cellular or other non-protein biologic intervention. Seven studies investigated repurposed or non-repurposed drugs for non-therapeutic effects in GBM such as symptom management. Nine trials were excluded as they examined completely novel therapies. A figure detailing key mechanisms of action of repurposed drugs investigated at the preclinical stage is presented in Figure 1. The data are also presented more comprehensively in Table 1. In Figure 2, the pathways targeted by repurposed drugs being investigated at the clinical stage are presented (also shown in Table 2).

## 3. Discussion

### 3.1. Repurposed Agents in Preclinical Study

#### 3.1.1. Antiarrhythmics

Interest in cardiac glycosides such as digoxin, and its distant relative proscillaridin A, for the treatment of glioma developed following discovery of their antineoplastic properties [18,20,159]. Primarily known for use in heart failure and arrhythmia, these agents were identified using a systems biology approach by integrating genomic profiles [159]. Digoxin, at clinically appropriate concentrations, was subsequently examined in vivo and shown to prolong survival in patient-derived xenograft (PDX) GBM models. However, given the narrow therapeutic index of digoxin, its potential for clinical use in patients is unclear [19]. Proscillaridin A also demonstrated antitumor effects in GBM both in vitro and in orthotopic xenografts via a mechanism involving glycogen synthase kinase (GSK) 3β activation and microtubule instability [20]. These agents have not been examined clinically.

#### 3.1.2. Antibiotics-Tetracyclines, Macrolides, and Antimycobacterials

Several antibiotics were examined as potential antineoplastics in GBM after they were found to demonstrate efficacy in malignancies outside of the central nervous system (CNS) [21,22,23]. Among these, the tetracyclines were identified as possible antineoplastics due to their inhibition of mitochondrial biogenesis, a feature central to GBM pathobiology [22]. Doxycycline induced mitochondrial dysfunction, oxidative stress and ultimately energy deficiency in multiple GBM cell lines [21,22]. Additionally, doxycycline was reported to behave synergistically in combination with TMZ, suggesting that it may act as a chemosensitizer [21]. Dapsone, an older antibiotic originally used for mycobacterial infections, was shown to decrease migratory activity and anchorage-independent growth in vitro, both of which are important features of GBM aggressiveness [23]. Clofazimine, another antimycobacterial agent, inhibited the gap junction protein Cx46 and caused apoptosis, decreased self-renewal and decreased tumor growth of glioma stem-like cells (GSCs) in combination with TMZ [24]. Despite these findings, it should be noted that BBB penetrance of clofazimine is limited. Although these agents remain to be studied clinically, their investigation may be warranted given their well-known safety profile and preclinical efficacy.

#### 3.1.3. Antidiabetics

Various diabetic agents have been shown to have antineoplastic action including the meglitinide, repaglinide, which has exhibited promise in early investigations but has not yet been translated to clinical trial [25]. Repaglinide was identified through a computational model analyzing differential gene expression between long-term and short-term surviving GBM patients. Repaglinide was found to decrease B-cell lymphoma 2 (BCL-2), programmed death ligand-1 (PD-L1), and Beclin-1 levels, factors that promote GBM pathobiology. In addition to repaglinide, another well-known antidiabetic agent studied in GBM is metformin. Given that metformin has been extensively studied clinically, it is covered in detail in a later section.

#### 3.1.4. Antidepressants

A logical step in the repurposing search for GBM therapy was considering agents known to have high BBB penetrance such as antidepressants, antiepileptic drugs (AEDs), and antipsychotics [26,96]. The observation that there is lower incidence of GBM in patients receiving antidepressant therapy further supported investigation of these agents [26]. Using a computational drug screen, tricyclic antidepressants (TCAs) such as imipramine were identified as a family of agents that may have anti-GBM action [16,26]. Mice bearing intracranial GBM treated with imipramine had increased survival as a result of tumor autophagy [26]. Moreover, combining imipramine with ticlopidine, an inducer of autophagy, further increased mouse survival with increased cAMP levels as well as dysregulation of autophagic regulatory gene 7 (ATG7). Another TCA, clomipramine, proved to be efficacious in treating GBM cells that have a phenylalanine for leucine mutation in the mitochondrial respiratory chain complex III cytochrome b subunit, a mutation enriched in GBM [27].

Selective-serotonin reuptake inhibitors (SSRIs) have also been identified as having anti-GBM potential [28]. Fluvoxamine, an SSRI used in the USA for obsessive-compulsive disorder and in other countries for major depression, was found to interfere with actin polymerization and thus attenuate the invasiveness of GBM cells [28]. Fluvoxamine decreased GBM cell migration, ruffle formation and invasiveness through interaction with the Protein kinase B (AKT)/mammalian target of rapamycin (mTOR) pathway. Additionally, in vivo experiments with intracranial xenografts showed decreased invasion and increased animal survival following treatment with fluvoxamine [28]. Another SSRI, sertraline, when combined with pterostilbene, a relative of the dietary supplement resveratrol, reduced cell viability, sphere formation and migration due to synergistic mitogen-activated protein kinase (MAPK) inhibition [29]. Although numerous studies highlight how antidepressant medications influence cell migration, growth and invasiveness, these agents remain to be investigated clinically.

#### 3.1.5. Anti-Inflammatories

Non-steroidal anti-inflammatory drugs (NSAIDs) are a class of agent used for everyday complaints and serious systemic diseases [160]. These drugs are generally well tolerated and affordable, attributes that make them excellent repurposing candidates. In addition, given that the immune response plays an central role in cancer promotion, anti-inflammatory/immunosuppressive medications target pathways that are critical to tumor pathobiology [161]. In this section, we will review the agents that have only been examined in the preclinical setting. Later, the few agents that have been translated to clinical analysis will also be discussed.

IP1867B, a combination of aspirin, triacetin and saccharin has substantial preclinical evidence supporting its use as an anti-GBM agent. IP187B inhibits IL6/signal transducer and activator of transcription 3 (STAT3), NF-κB, and insulin-like growth factor receptor pathways and has demonstrated additive efficacy when combined with epidermal growth factor receptor (EGFR) inhibitors against primary GBM cells and intracranial GBM xenografts [28,30,31,32]. Aspirin itself has also been investigated as an adjunct with doxorubicin in several cancer cell lines [33]. Specifically, it was reported that aspirin induced a more active immune TME including upregulating PD-1. Furthermore, when combined with anti-PD-1 therapy, there was increased tumor regression in multiple solid cancers [33]. Celecoxib was also shown to affect epithelial-mesenchymal transition (EMT) and attenuate GBM cell migration and proliferation [34]. While COX-2 inhibition has been considered the primary mechanism for the antineoplastic effects of celecoxib, deregulation of the NF-κB pathway may also play an important role in its antineoplastic properties against GBM and other malignant cells [35,36]. Celecoxib has also been examined clinically in GBM as detailed later.

The phosphodiesterase inhibitor, ibudilast, inhibits macrophage migratory inhibitory factor (MIF), a protein upregulated in a subset of MGMT methylated GBM tumors that have worse prognosis [37]. Ibudilast treatment suppressed proliferation of GBM cells and synergistically prolonged survival when combined with TMZ in PDX models [37]. Targeting the immune system from another angle is the disease-modifying antirheumatic drug (DMARD), sulfasalazine, which increases levels of reactive oxygen species (ROS) and induces apoptosis in combination with RT [38]. When sulfasalazine was examined in combination with radiosurgery, it synergistically prolonged survival of mice bearing intracranial GBM xenograft [38]. Due to the well-known chemical profile of sulfasalazine, a Phase I/II study was conducted in 10 patients with anaplastic astrocytoma or GBM with a primary endpoint of toxicity and response [39]. Unfortunately, the study was halted at the interim analysis due to a lack of improved clinical response and the development of toxicity in all patients including grade IV toxicity. In conclusion, despite their preclinical success, several NSAIDs such as ibudilast or sulfasalazine remain to be adequately studied clinically.

#### 3.1.6. Immunosuppressants

First-generation mTOR inhibitors such as everolimus, sirolimus and temsirolimus are a class of immunosuppressive agent and have undergone several investigations for GBM treatment [40,41,42]. While the preclinical data are presented here, these agents have also been studied clinically, as discussed in greater detail later. These agents act by a variety of mechanisms in addition to inhibition of mTOR such as targeting myeloid cell leukemia-1 (MCL-1) [40]. Although monotherapy with everolimus did not change MCL-1 protein abundance in vitro, combination therapy with a second mTOR inhibitor, or with olparib a poly-ADP ribose polymerase inhibitor, significantly decreased MCL-1 [40]. These combination regimens increased apoptosis and reduced growth of U87 GBM xenografts, findings thought to occur through a decrease in homologous recombination (HR) repair. Consistent with this, combining sirolimus with chloroquine and TMZ resulted in increased apoptosis in GBM cell lines [40,41], a finding likely due to an increase in DNA damage because of decreased HR repair and activation of the apoptotic cascade.

#### 3.1.7. Antihypertensives

Using high-throughput, cell-based drug screens, several antihypertensive and cardiovascular agents were identified as possible repurposing agents, although the majority of these remain to be studied in the clinic [18,162]. Mibefradil, a non-selective calcium channel blocker, inhibited the non-homologous end-joining (NHEJ) repair system and the T-type calcium channel, CAV3.2, a molecule upregulated in certain GBM patients that have worse prognosis [43]. The inhibition of NHEJ and CAV3.2 decreased growth and stemness through activation of apoptotic pathways and attenuation of AKT/mTOR signaling. Despite this, repurposing mibefradil is likely limited given its poor side-effect profile and potential drug interactions [163]. Another antihypertensive that was identified through targeted screening against monoamine receptors is prazosin, an alpha-1 adrenergic blocker originally used for hypertension and post-traumatic stress disorder. Prazosin induced apoptosis of GSCs and prolonged survival in an orthotopic GBM model [32]. Unfortunately, it has not yet been studied in the clinic. Amlodipine, a dihydropyridine calcium channel blocker, and pentoxifylline, a xanthine derivative used in peripheral arterial disease, also inhibited growth of GBM cells [44,45]. However, these agents have not undergone any meaningful clinical study. In conclusion, several antihypertensives have demonstrated promising preclinical findings that suggest they may have clinical efficacy, but these results remain to be recapitulated in the clinic.

#### 3.1.8. Antipsychotics

Antipsychotics are attractive antiglioma agents because of their propensity to cross the BBB and several antipsychotics have been identified as possible GBM therapies [46,55]. Thioridazine is a first-generation phenothiazine that has been extensively studied preclinically. It increased autophagy and upregulated AMP-activated protein kinase (AMPK) and microtubule-associated protein 1A/1B-light chain 3 phosphatidylethanolamine conjugate (LC3-II) [47]. Moreover, thioridazine also increased autophagy in flank xenografts, resulting in WNT pathway alterations that promoted β-Catenin degradation, autophagy and apoptosis [46,47]. Finally, thioridazine was found to act as a chemosensitizer of TMZ both in vitro and in an intracranial xenograft model [47,48]. Unfortunately, this agent has not yet undergone clinical investigation.

Other phenothiazine derivatives such as DS00329 or chlorpromazine have similarly had success in preclinical analysis. DS00329 decreased Cyclin A, B, and D1 protein abundance and increased p21, resulting in G1 phase arrest and apoptosis in U251 and U87 cells [49]. Contrastingly, chlorpromazine only demonstrated efficacy in TMZ resistant cells through inhibition of cytochrome c oxidase (CcO, complex IV) activity. This inhibition significantly prolonged survival in an intracranial GBM model, which was hypothesized to occur due to increased binding of chlorpromazine to CcO subunit 4 isoform 1 (COX4-1), the predominant isoform in chemoresistant cells [50,51].

Fluspirilene and penfluridol, both first-generation diphenylbutylpiperidine antipsychotics, were also found to impair GSC function [52,53]. Fluspirilene inhibited STAT3, resulting in decreased GBM proliferation, invasion and tumor growth, penfluridol decreased colony sphere number and size with reductions in (sex determining region Y)-box 2 (SOX2) and octamer-binding transcription factor 4 (OCT4) [52,53]. Additionally, penfluridol decreased migration through integrin interference and uPAR reduction and blocked EMT by attenuating ZEB1 expression. Notably, the combination of penfluridol and TMZ resulted in synergistically prolonged survival in mice bearing orthotopic xenografts.

Atypical antipsychotics have also demonstrated promise as possible repurposing anti-GBM agents [54,55]. In vitro assays demonstrated that olanzapine suppressed growth and migration of GBM cell lines [54]. When coupled with TMZ, olanzapine demonstrated a substantial chemosensitizing effect. Quetiapine had some promising results in orthotopic mouse GBM models both as monotherapy and in combination with TMZ, even against TMZ-resistant GBM [55]. Similar to the phenothiazine antipsychotics, quetiapine inhibited the WNT signaling pathway and promoted oligodendrocyte-like differentiation [47,55].

Importantly, there has been concern regarding the possible dose-limiting toxicities and side-effect profiles of some of the aforementioned antipsychotics. Given this, newer antipsychotics such as brexpiprazole are also under investigation as anti-GBM agents. Early results using brexpiprazole have shown cell growth inhibition and induction of apoptosis through reduction of survivin [56]. Overall, as a class, the antipsychotics have achieved significant preclinical success warranting translation to the clinic, where careful examination of their side-effect profile and drug interactions will be important.

#### 3.1.9. Antivirals

Antiviral medications have undergone limited preclinical investigation as anti-GBM agents; however, some evidence exists for their potential efficacy. Simeprevir, a Hepatitis C (HCV) antiviral, inhibits phosphatidylinositol 4-kinase (PI4K) IIIα, a factor thought to play a role in radiation resistance [57,58]. Simeprevir decreased phosphorylation of protein kinase C (PKC) and AKT and delayed γH2AX focus formation, resulting in apoptosis and autophagy [57]. Ribavirin, another HCV antiviral, decreased glioma cell growth and migration in vitro by targeting the eukaryotic initiation factor, EZH2, and extracellular signal-regulated kinase (ERK) pathways [58]. Additionally, combining ribavirin with chemotherapy and/or RT resulted in synergistically prolonged survival in several orthotopic GBM models. These translational findings support further analysis of this drug class clinically.

#### 3.1.10. Biologics and Small-Molecule Inhibitors

Large-scale molecular profiling of GBM such as The Cancer Genome Atlas (TCGA), have facilitated the identification of the pathways active in GBM that can be targeted by specific inhibitors [164,165]. Many small-molecule inhibitors are now being investigated in GBM that were initially investigated in other malignancies [59,60,61,62,63,97,148,164,166]. These drugs will only be discussed in brief as their antineoplastic mechanisms are generally the same as their originally intended mechanism of action.

Several small-molecule inhibitors, with a wide array of original indications, never made it to market for their initially developed indication. For example, a c-Jun N-terminal kinase (JNK) inhibitor, AS602801, originally developed for endometriosis, demonstrated cytotoxicity against neoplastic cells including GBM in vitro [59]. CEP-1347, another JNK inhibitor originally developed as an anti-Parkinsonian drug, was subsequently found to induce differentiation, decrease self-renewal and decrease tumor initiation in GBM cell lines [60]. These drugs remain to be studied clinically.

One of the most frequently altered pathways in GBM is the AKT/phosphoinositide 3-kinase (PI3K) signaling response, reported to be altered in almost 90% of these tumors [167]. Several small molecules target this pathway and have been examined in GBM. Inhibition of PI3K with LY294002 or PX-886 blocked the growth of GBM cells in combination with gamitrinib, a mitochondrial heat shock protein (HSP)-90 inhibitor [61]. Ibrutinib, a well-described tyrosine kinase (TK) inhibitor that targets the BMX-STAT3 pathway, suppressed the growth of GSCs and sensitized them to RT [62]. This inhibitor, in combination with TMZ, also prolonged survival of mice bearing intracranial GBM relative to TMZ alone. Roscovitin, another small-molecule inhibitor targeting a wide range of cyclin-dependent kinases (CDKs), was reported to act as a chemosensitizer to TMZ in an orthotopic GBM xenograft model [63]. Finally, the MEK inhibitor, binimetinib, was investigated in a non-randomized open-label clinical study in 62 patients with encorafenib, a BRAF inhibitor, in patients with recurrent BRAF V600-mutated high-grade glioma (HGG) [64]. While primary outcome is tumor radiographic response per response assessment in neuro-oncology (RANO) criteria, no results are currently available.

#### 3.1.11. Disulfiram

Disulfiram initially came to the attention of researchers as a possible adjunctive GBM therapy because of its ability to inhibit the aldehyde dehydrogenase enzyme family, a proposed biomarker for self-renewing cells in tumor populations [65]. It was theorized that targeting aldehyde dehydrogenase with disulfiram may interfere with GBM self-renewal. Subsequent investigation found that disulfiram inhibited self-renewal and growth of TMZ resistant GBM cells through inhibition of polo-like kinase 1 (PLK1), while minimally affecting normal astrocytes [65]. Disulfiram was also found to inhibit MGMT activity in GBM cells [66]. Various disulfiram formulations decreased MGMT abundance in GBM cells via the ubiquitin–proteasome pathway by modifying a cysteine residue, resulting in an increase in alkylator-induced cytotoxicity [66]. These findings were supported by findings in animal models where decreased MGMT activity was seen in the liver and brain.

Disulfiram was also noted to have enhanced cytotoxic effects in the presence of copper (Cu). Consequently, Cu–disulfiram metal complexes were developed [67]. These Cu–disulfiram complexes when combined with TMZ inhibited GSC growth and prolonged survival of mice bearing intracranial PDXs. The increase in survival was thought to have occurred due to alterations in DNA repair [66,67]. Despite these findings, the instability of the Cu–disulfiram compound and potentially low therapeutic efficacy raised questions regarding its use in patients. To resolve these issues, disulfiram based nanoparticles (termed passively-targeted DSF nanoparticles (DSFNPs)) were constructed [68]. DSFNPs increased disulfiram drug uptake within the CNS and prolonged disulfiram half-life in vivo. Additionally, DSFNPs demonstrated high cytotoxicity in vivo against intracranial glioma xenografts as a result of ROS generation and release of apoptosis inducing factor (AIF). Notably, DSFNPs induced mild regression of intracranial medulloblastoma xenografts in comparison to unencapsulated disulfiram, suggesting that use against GBM might be feasible. These preclinical studies have lead to several clinical trials that are discussed later.

#### 3.1.12. Methylxanthines

Methylxanthines, including theophylline, theobromine and caffeine, have undergone extensive investigation as anti-GBM therapies with mixed results. These findings have been reviewed in greater detail elsewhere [69]. Briefly, the main mechanism by which these agents act is via phosphodiesterase (PDE) inhibition. However, they also influence other signaling and epigenetic pathways crucial to tumorigenesis. Overall, these agents appear to be potentially promising candidates.

#### 3.1.13. Neurocognitive Agents

Active neurocognitive agents are attractive as they act within the CNS. Riluzole, an antiamyotrophic lateral sclerosis (ALS) drug, has undergone several mechanistic studies in conjunction with other agents but has not yet been translated to the clinic. Combining riluzole with imipramine, a TCA, and valproic acid (VPA), an AED, resulted in decreased GBM cell viability [70,71]. Riluzole also inhibited the internal ribosome entry site (IRES) trans-acting factor (ITAF) hnRNP A1, a factor involved in resistance to mTOR inhibitors [71]. By a similar mechanism, it was found that the combination of riluzole and sirolimus increased apoptosis in vitro and decreased growth in heterotopic murine GBM [71]. In addition to the above, riluzole is a thought to be a sodium channel blocker that inhibits glutamate release [72]. By this mechanism, riluzole was also shown to inhibit glucose transport 3 (GLUT3) in GSCs, resulting in inhibition of HIF1α and p-AKT signaling. Finally, riluzole was shown to downregulate DNA (cytosine-5-)-methyltransferase (DNMT1), a factor responsible for hypermethylation of tumor suppressors [72]. While riluzole has not been examined in patients, clinical studies with mTOR inhibitors are discussed in detail later.

Dimethyl fumarate is currently used in relapsing-remitting multiple sclerosis, but was also found to act against GBM through ERK1/2 and AKT inhibition in GBM cell lines [73]. Dimethyl fumarate remains to be studied in large clinical trials. Idebenone, a Coenzyme Q_10_ analogue, primarily used for neurocognitive disorders such as Alzheimer’s disease, was also shown to have pro-apoptotic effects [74]. Both U373MG and U87MG cell lines had decreased cell viability after idebenone treatment, while co-treatment with TMZ and oxaliplatin resulted in increased cytotoxicity in U373MG cells.

#### 3.1.14. Statins

Statins are widely used agents with well-established toxicity profiles and pleiotropic effects that include potential antineoplastic action [75,76,77,78,168]. Statins were found to interfere with c-Myc through microRNA (miRNA) targeting [76]. Consistent with this, in medulloblastoma, lovastatin was found to increase miR-33b, resulting in inhibition of c-Myc [76]. In GBM, lovastatin was found to inhibit S-phase kinase protein (Skp2), an E3 ligase involved in tumorigenesis, and when combined with TMZ increased degradation of Skp2 in cells and xenografts [75]. Simvastatin was found to inhibit EGFR, fibroblast growth factor receptor (FGFR) and proto-oncogene tyrosine-kinase Src (c-SRC) [77], TKs that are highly active in GBM. Atorvastatin demonstrated therapeutic potential through inhibition of RAS prenylation thereby inhibiting growth and decreasing survival of multiple GBM cell lines [78]. In this study, co-treatment of flank GBM xenograft with atorvastatin and TMZ demonstrated synergistic inhibition of growth.

Despite the above observations, a retrospective study of over one thousand patients with HGG showed that statins, as well as NSAIDs, were not associated with increased progression free survival (PFS) or OS [168]. Additionally, after stratifying by WHO grade, there was no significant association between statins and PFS/OS, although aspirin was associated with increased PFS and OS in grade III glioma. It remains unclear whether statins have a potential role in GBM therapy.

#### 3.1.15. Other

Several other therapeutics that do not fall into a general class and that have limited preliminary data are undergoing initial experimental evaluation as repurposing candidates. Aurintricarboxylic acid, a topoisomerase inhibitor, was reported to chemosensitize TMZ in vitro by altering NF-κB signaling via TWEAK [79]. Other medications such as papaverine, a non-narcotic opium alkaloid, inhibited cell proliferation of U87 and T98G GBM cells [80]. Bacoside A, the bioactive component of a traditional plant-based medicine, increased phosphorylation of the calcium/calmodulin-dependent protein kinase IIA disrupting fluid balance and promoting GBM cell lysis [81]. Another agent, verteporfin, a benzoporphyrin-like drug, has only demonstrated inhibitory effects against GBM cells under specific conditions such as hypoxia [82]. Verteporfin significantly inhibited primary and immortalized cell lines under 1% oxygen conditions through a Yes-associated protein (YAP)-independent mechanism and increased ROS formation. Clomiphene, a selective estrogen receptor modulator (SERM), is another agent identified through virtual screening [83]. This drug was found to interfere with IDH1 in GBM. Specifically, in HT1080 cells and flank xenografts, clomiphene allosterically inhibited mutant IDH1^R132H^ decreasing tumor size and the ability of IDH1^R132H^ to reduce α-ketoglutarate [83]. In sum, the agents above highlight the possible anti-GBM actions that potentially exist in a variety of drugs that are routinely and rarely used for other indications. A comprehensive list of small-molecule inhibitors that have been very preliminarily studied in GBM is available in the following recent publications [169,170].

#### 3.1.16. Targeting the Tumor Microenvironment (TME)

GBM is made up of a complex of tumor cells and surrounding microenvironmental components that include inflammatory cells, microvasculature and extracellular matrix (ECM) proteins [171]. In addition to the malignant cells, the TME also plays a critical role in promoting GBM pathobiology, and targeting the TME (e.g., the immune compartment or the vasculature) is an important adjunct to GBM therapy [171,172]. Repurposing agents to specifically target the TME represents a potential novel avenue for GBM treatment. Perhaps the best-known repurposing strategy targeting the TME has been the use of bevacizumab to block angiogenesis. In GBM, following the success of several Phase II clinical trials examining bevacizumab [173,174], a Phase III trial was conducted demonstrating that addition of bevacizumab increased PFS without significantly altering OS [175]. Another agent targeting the TME is the antiparasitic mebendazole, an agent reported to inhibit the angiogenic factor, VEGFR2 [102,103,126,132]. Additionally, the benzoporphyrin-like drug, verteporfin, was reported to target the hypoxic TME in GBM to induce hypoxic tumor cell death [82].

Inflammatory cells represent another important component of the TME and have been targeted by a variety of agents including NSAIDs, mTOR inhibitors and checkpoint inhibitors. The use of these agents was comprehensively described in other subsections of this review.

The final component of the TME is the ECM. While targeting ECM factors such as laminin has been examined in other cancers, it has been poorly studied in GBM. In one study, pirfenidone, an antifibrotic agent, was shown to decrease the abundance of ECM components like collagen and hyaluronan to improve blood flow and anticancer agent delivery [176].

#### 3.1.17. Inhibition of Signaling Pathway Active in GBM

In examining drugs potentially useful for GBM therapy, it was reasoned that agents targeting the major pathways active in GBM represent a potentially important subset of anti-GBM drugs. The pathways most commonly activated in GBM include signaling downstream of receptor tyrosine kinases (RTKs); the PI3K/AKT/mTOR pathway; RAS/MAP/ERK signaling; TGF-B and NF-κB [177]. Many of the agents targeting these pathways have been extensively discussed throughout this review. The PI3K/AKT/mTOR response is potentially the most active pathway in GBM. Several repurposed agents inhibiting components of this pathway have been described in this review such as perifosine and fimepinostat [42,61,122].

#### 3.1.18. Targeting Glioma Stem Cells

Finally, it is important to note that repurposing has been used to specifically target cancer stem cells (or glioma stem cells-GSCs). While several drugs targeting GSCs have been mentioned, certain agents were identified specifically because of their propensity to target GSCs. Chlorpromazine is one such agent, an antipsychotic that was shown to decrease stemness markers and neurosphere formation [178]. Nicardipine, a calcium channel blocker used for hypertension, was shown to promote apoptosis and enhance the toxic effect of TMZ against patient-derived GSCs [179]. GSCs have also been shown to have a specific dependence on mitochondrial function, and mitochondrial inhibitors such as trifluoperazine, mitoxantrone or pyrvinium pamoate were reported to specifically kill GSCs [180]. These agents were shown to induce selective cytotoxicity of GSCs substantially more than TMZ. Doxazosin was also found to target GSCs and increase sensitivity to the EGFR inhibitor, osimertinib [181]. Finally, the anti-Parkinsonian, trihexyphenidyl, was repurposed to specifically target GSCs and shown to decrease GSC proliferation [182].

### 3.2. Repurposed Agents in Clinical Investigations

#### 3.2.1. Antiepileptic Drugs (AEDs)

Patients with GBM often have seizures during their disease course, resulting in the widespread use of AEDs in this population. Given this observation, AEDs that have antineoplastic effects represent a potentially fruitful source for repurposing [183]. VPA, a histone deacetylase inhibitor (HDACi) with cytotoxic activity in other cancer types, is the AED that has been most extensively studied in GBM [84]. In diffuse intrinsic pontine glioma (DIPG), the combination of VPA and carboplatin increased apoptosis through acetylation of Histone H3 [84]. Similarly, when combined with statins, VPA synergistic induced apoptosis [85]. On the other hand, VPA was reported to act in an additive fashion with tranylcypromine, a TCA, and riluzole, an agent used for ALS [70]. VPA also sensitized GBM cells to TMZ, while an alkylated hybrid of TMZ and VPA decreased viability and proliferation of GBM cells by promoting autophagy [86].

Based on these promising preclinical results, several groups examined VPA in clinical GBM. These trials have primarily used a combinatorial approach where VPA was combined with other repurposed agents or chemotherapeutics [87,88]. For example, in one Phase II study, VPA was used with RT or other biological agents such as bevacizumab [88]. The study began in 2009, enrolled 38 pediatric patients with GBM, anaplastic astrocytoma or gliosarcoma and recently concluded. No results are yet available. The primary outcome measure was event-free survival and VPA toxicity when combined with RT. Another trial investigates VPA with sorafenib, a multikinase inhibitor that has increased efficacy in the setting of HDAC inhibition [87]. This Phase II trial started in 2013 with plans to enroll 47 HGG participants; it will be completed in 2022. Its primary aim is to investigate whether the combination regimen improves PFS, while secondary outcomes include OS, best response rate and adverse events. Despite the strong preclinical evidence and safety, it remains to be determined whether VPA will be beneficial in patients with GBM.

#### 3.2.2. Disulfiram

As highlighted in the first section, there is a substantial amount of preclinical evidence supporting the repurposing of disulfiram for GBM. Several clinical trials have examined Cu–disulfiram or disulfiram alone in GBM [89,90,91]. In a Phase I study in GBM investigating disulfiram and TMZ following standard chemoradiotherapy, patients demonstrated dose-related toxicities in the higher disulfiram dose group (1000 mg vs. 500 mg daily) including grade III delirium, fatigue, ataxia and peripheral neuropathy [89]. The median OS in these patients was 5.4 months from the time of disulfiram initiation. The trial concluded that disulfiram can be safely combined with TMZ, but may result in reversible neurotoxicity. The results of this Phase I study prompted an open label, randomized Phase II/III study, which began in 2016, with a plan to enroll 142 GBM patients at initial recurrence [91]. The intended study design was a 1:1 randomization of patients to receive either Cu–disulfiram and alkylating chemotherapy or alkylating chemotherapy alone (lomustine, procarbazine, vincristine, or temozolomide). The trial is still enrolling patients and the results are pending. While disulfiram has shown promising results in early studies, it remains to be determined whether the efficacy will be recapitulated in randomized trials.

#### 3.2.3. Antifungals/Antimalarials

Antifungal agents have recently been identified as potential anti-GBM agents. Among this category of drugs, azoles have shown some potential for clinical translation [92,93]. The current understanding of the antineoplastic action of azoles involves their ability to inhibit hexokinase II leading to restriction of GSC proliferation and GBM growth [93]. Specifically, in experimental GBM, ketoconazole and posaconazole decreased metabolic activity leading to increased animal survival and decreased tumor growth [92]. These preclinical studies led to an ongoing open-label, non-randomized Phase I trial investigating posaconazole and ketoconazole in patients with recurrent HGG [94]. The trial began in 2018 with plans to enroll 30 patients with a primary outcome of intratumoral ketoconazole and/or posaconazole concentration. Clioquinol is another topical antifungal being investigated in GBM [92,95]. Together, these results suggest that although there is some preclinical evidence for the potential use of azoles and clioquinol for GBM, their clinical safety and efficacy remain to be determined.

Similar to the antifungal class, antimalarials have also been identified as having antineoplastic potential. Antimalarials, including chloroquine, mefloquine and atovaquone have all emerged as strong repurposing candidates although their use has been hampered by technical challenges [31,41,99,100,101]. Atovaquone was initially found to inhibit STAT3, but did not achieve high enough tissue levels to deliver a therapeutic effect in the CNS [31]. In an attempt to tackle this problem, an enhanced dispersion formulation was created which demonstrated increased cytotoxicity against GBM cells in vitro and in an animal GBM model [31]. Another antimalarial, chloroquine, was shown to interfere with GBM progression through autophagy and apoptosis [26,41,86,96]. A combination of chloroquine, sirolimus and TMZ resulted in apoptosis and decreased growth of intracranial GBM, while tri-therapy with chloroquine, crizotinib and sorafinib was efficacious against 3D patient-derived cell cultures [41,97]. These preclinical results prompted a non-randomized, open-label Phase I/II clinical trial which began in 2019 and plans to enroll 75 patients with either low-grade glioma (LGG) or HGG investigating co-administration of hydroxychloroquine, dabrafenib, a BRAF inhibitor, and trametinib, a MEK inhibitor [98]. The trial has a primary endpoint of maximum tolerated dose (MTD) and clinical response in the Phase II portion of the trial. The Phase II trial plans to enroll 62 participants with recurrent HGG with a primary endpoint of tumor radiographic response and secondary outcomes of PFS and OS.

Antimalarials have also been examined in conjunction with other repurposed agents. The combination of mefloquine with metformin and memantine was recently investigated in a Phase I/II clinical trial in newly diagnosed GBM patients receiving traditional TMZ [99,100,101]. The results of this trial were recently published. The most common adverse effect was lymphopenia, while OS from study entry was 21 months with a 43% two-year OS. The conclusion from this study was that tri-therapy is safe in combination with TMZ in newly diagnosed GBM. Although there was no comparison group and the study was not powered to study efficacy, the results appeared promising as a possible component of future multipharmacologic therapy.

It is important to note that antimalarials have side-effects that may be a significant barrier to their use in GBM. [184,185]. Specifically, while nausea, vomiting and diarrhea can restrict their use, neurotoxicity has also been reported. In addition, their immunomodulatory actions might also effect their efficacy against GBM.

#### 3.2.4. Antiparasitics

The antiparasitic, mebendazole, was recently identified as a potential antineoplastic agent in medulloblastoma and was subsequently examined in GBM [102,103]. Mebendazole inhibits vascular signaling by competing with ATP for binding vascular-endothelial growth factor 2 (VEGF2), resulting in decreased tumor angiogenesis and microvascular density in murine medulloblastoma. Mebendazole also inhibits microtubule formation leading to decreased tumor burden via metaphase arrest [104,105]. A Phase I study investigating oral mebendazole opened in 2016 and will enroll 21 patients with recurrent or resistant-to-standard-therapy tumors including pediatric GBM [106]. As a non-randomized, open-label trial, it will primarily analyze adverse events. This trial will conclude in 2022.

#### 3.2.5. Antihypertensives

In the United States, angiotensin-converting enzyme (ACE) inhibitors and angiotensin II receptor blockers (ARBs) make up a large percentage of prescribed antihypertensives. They act by interfering with the renin-angiotensin-aldosterone-system (RAAS). Their well-known safety profile and low cost make them excellent candidates for repurposing [107]. Although these agents have undergone some degree of clinical investigation, they remain to be studied in randomized controlled trials [32,44,45,107]. A recent analysis of two large clinical studies in GBM, comprising a total of 810 patients, was investigated by stratifying patients by their use of ACE inhibitors, ARBs or statins while in the trial. No significant benefit with regard to PFS and OS was seen for any of the drug classes. Of course, these clinical trials were not specifically designed to study ACE inhibitors or ARBs. Interestingly, ARBs were studied in a randomized trial as a possible adjunct to reduce steroid use by decreasing peritumoral edema [108]. In this trial, known as the ASTER trial, researchers looked to see if losartan could reduce the steroid requirement during RT in patients with newly diagnosed GBM. In the 75 patients enrolled, it was determined that losartan was not effective at reducing steroid requirement, although it was well tolerated. Secondary analyses also revealed that losartan had no impact on OS alone or when stratified by MGMT status. Finally, the ACE inhibitor, captopril, was studied as a component of a combination treatment trial that is discussed in further detail below. The above observations indicate that in non-randomized retrospective analyses these antihypertensives have not yielded fruitful results. It is unclear whether a significant effect would be observed in a more controlled setting.

#### 3.2.6. Anti-Inflammatories and Immunosuppressants

As noted earlier, mTOR inhibitors are immunosuppressive agents that range in their clinical indications from inflammatory bowel disease to dermatological disease and transplant rejection. Preclinical evidence for mTOR inhibitors as antiglioma agents is plentiful (see above). Temsirolimus was investigated in a Phase I study in GBM in combination with perifosine, a novel AKT/PI3K inhibitor, with primary outcomes of safety and determining Phase II dosing [42]. The trial began in 2014 and was completed in 2017. Although the trial found the combination was tolerable in 35 patients, no Phase II study has been conducted. Another mTOR inhibitor, everolimus, is being investigated in multiple Phase I and Phase II studies in GBM in combination with repurposed small-molecule inhibitors such as sorafenib, ribociclib and dasatinib [112,113,114,115,116]. One of these studies is a non-randomized, open-label Phase I study which planned to enroll 24 participants who have DIPG or HGG using the combination of ribociclib and everolimus to determine MTD [112]. The trial began in 2017 and was completed in July 2020. No results are yet available. Another non-randomized Phase I study with a goal of 24 recurrent GBM participants opened in December 2018 and examines OS and PFS in patients receiving ribociclib and everolimus [114]. An additional single-institution combined Phase I–II trial conducted by the National Cancer Institute that started in April 2016 using everolimus with sorafenib has a target enrollment of 118 patients who have recurrent HGG and primary outcomes of MTD and PFS [113]. Finally, a Phase II study that will investigate dasatinib with everolimus in a single group assignment of 24 patients including both recurrent or newly diagnosed DIPG or HGG, began in December 2017 and is estimated to be completed in December 2023 [115]. Primary outcome measures of the study include PFS and overall response rate (ORR). In sum, despite the preclinical excitement of mTOR inhibitors, their efficacy has not yet been proven in the clinical setting.

Celecoxib, an NSAID and selective COX-2 inhibitor, has been investigated in multiple clinical trials [109,110,111]. A Phase II trial examined 37 patients treated with the combination of irinotecan and celecoxib in recurrent malignant glioma patients with a primary endpoint of radiographic response and secondary endpoints of PFS, OS and safety [109]. Six patients achieved radiographic response and 13 achieved stable disease following treatment. Another Phase II study using TMZ, thalidomide and celecoxib in a cohort of 50 newly diagnosed GBM patients found that although the combination was well tolerated, no benefit in the primary outcome of PFS at four months was seen [110]. In a different Phase II trial, celecoxib was examined with 13-cis-retinoic acid in 25 patients with recurrent GBM [111]. The combination was found to have no greater effect on PFS than 13-cis-retinoic acid alone although the combination was well tolerated. Based on the results of these Phase II studies, celecoxib has not recapitulated the success seen in preclinical models, suggesting that it is likely not a clinically useful adjunct for GBM treatment.

#### 3.2.7. Antineoplastics

The topic of antineoplastic repurposing will only be briefly discussed given that the mechanisms of action of these drugs against GBM are the same as their original hypothesized mechanism. Many of these agents have been investigated preclinically including hydroxyurea, actinomycin D, carmofur, eribulin and paclitaxel [186,187,188,189,190,191,192,193,194,195]. With regard to clinical trials, vorinostat, an antineoplastic agent used for cutaneous T-cell lymphoma, was studied in a Phase I study in combination with TMZ [117]. This study began in 2005 and ended in 2011 demonstrating that the combination was well tolerated and led to a Phase I/II study that concluded there was no increase in the primary endpoint of PFS in 39 patients. Although it was suggested that the lack of increased PFS was due to a heavily pretreated group, further investigations have not been reported.

Other antineoplastic agents include cabozantinib, a TK inhibitor being investigated in a Phase II study of 10 pediatric patients with GBM or other HGG with a primary endpoint of disease response at six months [118]. Arsenic trioxide, an inorganic compound used to treat leukemia, is being investigated in a Phase I/II study as adjuvant therapy with TMZ and RT in a group of 50 participants with GBM, AA or other primary pediatric CNS tumor [119]. Marizomib, a proteasome inhibitor, has been added to standard chemoradiation therapy, TTF or in combination with bevacizumab in different Phase I studies [120,121]. One of these trials started in 2016 and recruited a cohort of 66 newly diagnosed GBM patients. The trial has recently concluded and at the time of writing of this article, the study results were unavailable. Primary outcome measures include adverse events and marizomib MTD. Fimepinostat, a PI3K inhibitor and HDAC inhibitor that originally received orphan drug designation for diffuse large B-cell lymphoma, is also under investigation in combination with standard treatments to confirm BBB permeability [122]. The study began in 2019 with a goal of recruiting 30 patients with DIPG, recurrent GBM or other primary CNS pathology and will assess primary outcome of fimepinostat BBB penetration. In conclusion, although a variety of antineoplastic agents have been repurposed for GBM therapy, it remains unclear whether their success in other cancers will show similar results in GBM.

#### 3.2.8. Carbonic-Anhydrase Inhibitors

Through examination of patterns of chemoresistance, novel repurposing agents have been identified such as acetazolamide. Acetazolamide, a carbonic anhydrase (CA) inhibitor, was reported to improve the cytotoxic effect of TMZ despite lacking any cytotoxic effect on its own. It was also found to augment the efficacy of TMZ in PDX models of GBM [123,124]. Mechanistic studies suggested that this response involved inhibition of carbonic anhydrase II (CAII) downstream of the proto-oncogene, BCL-3, and that this pathway promotes chemoresistance in patients with a methylated MGMT promoter [123]. Based on promising preclinical results, acetazolamide was investigated in a Phase I study as an adjunct to TMZ. This study began in 2018 and plans to conclude by 2022 [125]. The trial will enroll 60 patients with newly diagnosed, MGMT methylated GBM to evaluate the primary endpoint of toxicity and the secondary outcomes of OS, PFS and ORR. As part of the study, secondary outcomes will be compared to intratumoral BCL-3 abundance.

#### 3.2.9. Checkpoint Inhibitors

Immune checkpoint inhibitors function as a class of biologics that interact with the immune system to encourage an antitumor response by immune cells. This may mean interfering with antidestruction signals upregulated by tumor cells, or by altering the immune microenvironment to encourage immune cell infiltration. Two of the most-commonly used checkpoint inhibitors, nivolumab and pembrolizumab, interfere with the programmed cell death pathway. These drugs were originally developed for other oncological diseases but were recently repurposed as potential anti-GBM therapeutics.

Nivolumab, a monoclonal antibody targeting the interaction between programmed cell death 1 (PD-1), and its ligand PD-L1, has already undergone extensive clinical study in GBM. A Phase II study completed in 2017 investigated neoadjuvant nivolumab in 30 primary or recurrent GBM patients with a primary outcome of percent change in PD-L1 expression in tumor cells and lymphocytes following neoadjuvant nivolumab [127]. Secondary outcomes included RANO response rate and safety. Treatment was well tolerated, OS was 7.3 months and resected tumors demonstrated upregulation of immune pathway components [128]. Following demonstration of safety and tolerability in an initial Phase I trial, the Checkmate 143 Phase III open label, randomized trial was subsequently conducted comparing nivolumab against bevacizumab in 369 patients with recurrent GBM [126,129]. The primary endpoint of OS demonstrated that nivolumab was comparable to bevacizumab, suggesting that nivolumab monotherapy did not improve OS in recurrent GBM. Other clinical trials investigating these agents include Checkmate 498, a Phase III trial enrolling 550 newly diagnosed, MGMT unmethylated GBM patients comparing nivolumab to TMZ both in combination with RT. Unfortunately, it was recently announced that the primary outcome of OS was not met [130]. Checkmate 548 was another Phase III trial investigating 550 patients with newly diagnosed, MGMT methylated GBM comparing nivolumab to TMZ both with RT [131]. Again, nivolumab demonstrated no increase in OS.

Pembrolizumab is also under investigation in a variety of Phase I studies [133,134]. One of these studies aims to enroll 110 participants in a single group with primary endpoints of adverse events, sustained objective response and percentage of PD1+ CD8+ T cells in peripheral blood after 6 weeks of pembrolizumab therapy [133]. In another Phase I study, pembrolizumab was added to RT and bevacizumab in 32 patients with glioma with the primary endpoint of MTD, preliminary results demonstrated safety. Subsequently, pembrolizumab was investigated in a Phase II study with or without concurrent bevacizumab in 80 GBM patients with a primary outcome of PFS at six months [132]. Notably, PFS was 26% at six months in pembrolizumab alone compared to 6.7% at six months when pembrolizumab was combined with bevacizumab.

Overall, as a class, although checkpoint inhibitors initially appeared to have therapeutic benefit, this has not been recapitulated in large randomized trials. Nevertheless, the data have raised important questions regarding the role of immunotherapy and have spurred new trials using these agents in novel ways such as in the neoadjuvant setting or in specific sub-cohorts of patients [196,197].

#### 3.2.10. Diabetic Agents

Epidemiological studies previously noted decreased incidence, and possibly favorable outcome, for GBM patients actively taking metformin, a diabetic biguanide drug [135,136]. Metformin has potential antineoplastic mechanisms that include chloride channel inhibition, AMPK activation and mTOR interference [46,135]. Treatment of human GSCs with metformin induced G1 phase arrest and promoted apoptosis [135,137]. Other biguanide derivatives similar to metformin were also found to inhibit GSC proliferation and xenograft growth [138].

These preclinical findings suggested that metformin might be effective in patients and the use of metformin was analyzed retrospectively in GBM cancer registries [139,140]. In a retrospective analysis of 1093 patients with HGG, metformin was linked to increased OS and PFS in patients with grade III glioma, while no benefit was found in grade IV tumors [139]. It was speculated that this was due to the greater proportion of IDH mutations in grade III tumors. Consistent with this, in a second retrospective analysis of 1,731 patients, no beneficial effect was observed with metformin. However, if patients were taking metformin prior to their glioma diagnosis, they had a significant survival benefit in comparison to those who began the drug after diagnosis [140].

Recently, a Phase Ib/II study of 18 patients with various solid tumors including GBM was completed with a planned 3 + 3 drug escalation scheme to evaluate metformin MTD [141]. Results from this study are still pending. Another Phase I clinical trial is investigating metformin in combination with TMZ [142]. This trial began in 2011 and is estimated to conclude in 2022 with a plan to recruit a total of 144 GBM patients with a primary endpoint of safety and tolerability. Several other ongoing smaller clinical trials also look at metformin in GBM, although it remains unclear whether these trials have the cohort size necessary to evaluate efficacy [143,144,145].

Beyond these preliminary studies, a Phase II study enrolling 50 GBM patients started in 2015 and examined two-weeks of neoadjuvant metformin and TMZ followed by RT with a primary outcome measure of OS [146]. This study will likely be completed in 2022. Another Phase II study comprised of 108 GBM patients randomized to either TMZ plus metformin or TMZ and placebo began in 2016 and will conclude soon [147]. The primary outcome measure is PFS, with secondary measures of response rate, six month survival and overall quality of life. While metformin has promising preclinical data and possible therapeutic benefit in retrospective cohorts, it remains to be determined whether this will be recapitulated in randomized controlled trials.

#### 3.2.11. Other Clinical Use of Small Molecules and Biologics

In addition to direct antineoplastic effect, biologic agents have also been repurposed as imaging aids [148]. Cetuximab, an EGFR inhibitor, was used as a fluorescently labeled dye delivered at the time of surgery as an intraoperative guide to improve tumor resection [148]. 5-aminolevulinic acid (ALA) is perhaps the best known fluorescent agent that was studied as an intraoperative adjunct for tumor resection in GBM [149,150,151,152]. A phase III trial published in 2006, demonstrated that intraoperative 5-ALA fluorescence in HGG patients allowed for more complete resections and better outcomes in comparison to traditional microsurgery [152].

#### 3.2.12. The Cocktails

A number of multidrug regimens that involve using repurposed agents with standard treatments are being investigated for the treatment of GBM. These regimens target multiple pathways in the hope of inducing a greater overall effect than with monotherapy [153,154,155,158,198]. Some of these combinations have transitioned to clinical trials, while others remain in the preclinical phase.

One cocktail is the coordinated undermining of survival paths (CUSP) regimen that uses nine drugs: aprepitant, auranofin, captopril, celecoxib, disulfiram, itraconazole, minocycline, ritonavir, and sertraline [5,153,155,156]. In vitro, the CUSP9 regimen in combination with TMZ had a greater effect than TMZ alone acting primarily via inhibition of the WNT pathway [155]. Additive effects were also seen when CUSP9 was combined with BCL-2 pathway inhibition [154]. Subsequently, CUSP9 was investigated in a proof-of-concept study involving 10 patients with GBM and metronomic TMZ with a primary endpoint of toxicity [157]. The trial is not currently recruiting and no results have been released.

Another cocktail, known as CLOVA, consists of cimetidine, lithium, olanzapine, and VPA, was specifically designed to inhibit the GSK-3β pathway. Preclinical work using a retrovirus-induced GBM model found that the cocktail with TMZ decreased cellular proliferation but did not statistically affect tumor size when compared to the control of TMZ alone [153]. The same study also examined seven patients with recurrent GBM, demonstrating safety. Finally, the combination of celecoxib, vinblastine and cyclophosphamide alternating with methotrexate in a metronomic fashion was examined in a heterogenous group of pediatric patients with brain tumors including five HGG and 11 LGG [158]. The results found that in the 11 patients with LGG, those who received the treatment combination did better in terms of disease stabilization, as defined in the protocol, compared to historical controls. In general, the available data suggest that combination cocktails have not been shown to significantly improve outcome above that seen with standard therapy, although no randomized studies have been conducted.

#### 3.2.13. Limitations

In many studies, there is a high risk of bias due to small sample size and the lack of control patients. However, for the purposes of a complete review, they were included for discussion. In addition, it is possible that some published studies were not captured by the search terms used. For clinical studies, the study size, primary endpoint, patient population, study type and intervention were documented to enable efficient and critical review.

## 4. Conclusions

Here, a comprehensive review of repurposing therapeutics for GBM is provided, highlighting many attractive candidates. While a large group of agents have yielded positive preclinical data, translation to the clinic has been modest. Of course, given that many of these agents have already been used clinically, Phase I safety studies have often had good results. Despite this, potential disadvantages to repurposing include the interaction of these agents with other drugs and concerns with regulatory and intellectual properties. Such consequences can potentially offset the cost savings of repurposing. It remains to be determined whether repurposed agents will in fact reveal therapeutic efficacy in randomized studies. General observations suggest that improved effects can be seen when repurposed agents are combined with each other or when added to the current standard-of-care regimens, suggesting that this should be considered when planning future trials. Finally, it is important to highlight the potential role of immunotherapy and cell-based therapies, such as CAR-T cell therapy, in the treatment of GBM [199,200]. These novel modalities represent the latest agents to have been studied in clinical trials. Although these strategies have not yet proven to be effective clinically, their potential remains exciting [201,202].

## Figures and Tables

**Table 1:** Drug class, specific drug, and drug targets of repurposed agents currently undergoing preclinical investigation.

Drug Class	Drugs	Targets	Reference(s)
Antiarrhythmics	Digoxin	Na+/K+ ATPase, AKT	[19]
	Proscillaridin A	GSK3β	[20]
Antibiotics	Tetracyclines	Mitochondria	[21,22]
	Dapsone	FPR, IL-8, Leukotriene-B4	[23]
	Clofazimine	Cx46	[24]
Antidiabetics	Repaglinide	BCL-2, PD-L1, Beclin 1	[25]
Antidepressants	Imipramine	ATG7	[26]
	Clomipramine	Complex III Cytochrome B	[27]
	Fluvoxamine	AKT/mTOR	[28]
	Sertraline	MAPK	[29]
Anti-inflammatories	IP187B/Aspirin	STAT3, NF-κB, IGFR, PD-1	[28,30,31,32,33]
	Celecoxib	COX-2, NF-κB	[34,35,36]
	Ibudilast	MIF	[37]
	Sulfasalazine	System X(c)(-) Antiporter	[38,39]
Immunosuppressants	Everolimus	mTOR, MCL-1	[40,41,42]
	Sirolimus		
	Temsirolimus		
Antihypertensives	Mibefradil	NHEJ, Cav3.2	[43]
	Prazosin	AKT	[32]
	Amlodipine	PKD, Caspase 3	[44]
	Pentoxifylline	NA	[45]
Antipsychotics	Thioridazine	AMPK, MAP1/LC3-II, WNT	[46,47,48]
	DS00329	Cyclin A, Cyclin B, Cyclin D1	[49]
	Chlorpromazine	CcO Complex IV	[50,51]
	Fluspirilene	STAT3	[52]
	Penfluridol	SOX2, OCT4, uPAR	[53]
	Olanzapine	AMPK	[54]
	Quetiapine	WNT	[55]
	Brexpiprazole	Survivin	[56]
Antivirals	Simeprevir	PI4K	[57]
	Ribavirin	EZH2, ERK	[58]
Biologics and Small-Molecule Inhibitors	AS602801	JNK	[59]
	CEP-1347		[60]
	LY294002	PI3K	[61]
	PX-886		[61]
	Ibrutinib	TK, BMX-STA3	[62]
	Roscovitin	CDK	[63]
	Binimetinib	MEK	[64]
	Encorafenib	BRAF	[64]
Disulfiram	Disulfiram	PLK1, Ubiquitin–Proteasome Pathway, AIF	[65,66,67,68]
Methylxanthines	Theophylline	PDE	
	Theobromine		[69]
	Caffeine		
Neurocognitive	Riluzole	Na+ Transporter, ITAF hnRNP A1, HIF1A, AKT	[70,71,72]
	Dimethyl fumarate	ERK1/2, AKT	[73]
	Idebenone	p21	[74]
Statins	Lovastatin	c-Myc, SKP2	[75,76]
	Simvastatin	EGFR, FGFR, c-SRC	[77]
	Atorvastatin	RAS	[78]
Other	Aurintricarboxylic acid	NF-κB	[79]
	Papaverine	HMGB1/RAGE	[80]
	Bacoside A	CAMKIIA	[81]
	Verteporfin	YAP, HIF1A	[82]
	Clomiphene	IDH1	[83]

Abbreviations: protein kinase B (AKT); glycogen synthase kinase (GSK3B); formyl peptide receptor (FPR); interleukin-8 (IL-8); connexin 46 (Cx46); B-cell lymphoma 2 (BCL2); programmed death-ligand 1 (PDL1); autophagy related 7 (ATG7); mechanistic target of rapamycin (mTOR); mitogen-activated protein kinase (MAPK); signal transducer and activator of transcription 3 (STAT3); nuclear factor kappa-light-chain-enhancer of activated B cells (NF-KB); insulin growth factor receptor (IGFR); programmed cell death protein 1 (PD-1); cyclooxygenase-2 (COX2); macrophage migration inhibitory factor (MIF); induced myeloid leukemia cell differentiation protein (MCL-1); non-homologous end-joining (NHEJ); polycystin-1 (PKD); microtubule-associated protein 1A (MAP1)/1B-light chain 3 phosphatidylethanolamine conjugate (LC3-II); cytochrome c oxidase (CcO, complex IV); (sex determining region Y)-box 2 (SOX2); octamer-binding transcription factor 4 (OCT4); urokinase plasminogen activator surface receptor (uPAR); AMP-activated protein kinase (AMPK); phosphatidylinositol 4-kinase (PI4K); enhancer of zeste homolog 2 (EZH2); extracellular signal-regulated kinase (ERK); S-phase kinase protein (Skp2); epidermal growth factor receptor (EGFR); fibroblast growth factor receptor (EGFR); proto-oncogene tyrosine-protein kinase Src (c-SRC); rat sarcoma (RAS); high-mobility group protein 1 (HMG-1); receptor for advanced glycation endproducts (RAGE); Ca2+/calmodulin-dependent protein kinase II (CAMKII); isocitrate dehydrogenase 1 (IDH1).

**Table 2:** Drug class, specific drug, drug target, and current clinical phase of repurposed agents currently undergoing clinical investigation.

Drug Class	Drugs	Targets	Clinical Trial Stage	References
Antiepileptic Drugs	Valproic Acid	HDAC	Phase II	[70,84,85,86,87,88]
Disulfiram	Disulfiram	PLK1, Ubiquitin–Proteasome Pathway, AIF	Phase II/III	[89,90,91]
Antifungals	Azoles	Hexokinase II	Phase I	[92,93,94]
	Clioquinol	BAX, BCL-2	Phase I	[92,95]
Antimalarials	Atovaquone	STAT3	Preclinical	[31]
	Chloroquine	Unclear	Preclinical	[26,41,86,96,97]
	Hydroxychloroquine	LC3-II	Phase I/II	[98]
	Mefloquine	NMDA	Phase I/II	[99,100,101]
Antiparasitics	Mebendazole	Microtubules, VEGF	Phase I	[102,103,104,105,106]
Antihypertensives	ARBs, ACEis	Unclear	Retrospective	[32,44,45,107,108]
Anti-inflammatories	Celecoxib	COX-2	Phase I–II	[109,110,111]
Immunosuppressants	Temsirolimus	mTOR, MCL-1	Phase I	[42]
	Everolimus		Phase I/II	[112,113,114,115,116]
Antineoplastics	Vorinostat	HDAC	Phase I/II	[117]
	Cabozantinib	TK	Phase II	[118]
	Arsenic Trioxide	Cytochrome C	Phase I/II	[119]
	Marizomib	Proteasome	Phase I	[120,121]
	Fimepinostat	PI3K, HDAC	Phase I	[122]
Carbonic-Anhydrase Inhibitors	Acetazolamide	CA, BCL3	Phase I	[123,124,125]
Checkpoint Inhibitors	Nivolumab	PD-1	Phase II–III	[126,127,128,129,130,131]
	Pembrolizumab	PD-1	Phase I–II	[132,133,134]
	Ipilimumab	CTLA-4	Phase I	[129]
Diabetic Agents	Metformin	AMPK, Cl-Channels, mTOR	Phase I/II	[46,135,136,137,138,139,140,141,142,143,144,145,146,147]
Small Molecules and Biologics	Cetuximab	EGFR	Phase I	[148]
	5-ALA	Not Applicable	Phase III	[149,150,151,152]
Cocktails	CUSP-9	Survival Pathways	Phase I	[5,153,154,155,156,157]
	CLOVA	GSK-3B	Phase I	[153]
	Celecoxib, Vinblastine, Cyclophosphamide	COX-2, Microtubules, DNA	Phase I	[158]

Abbreviations: Histone deacetylase (HDAC); polo-like kinase 1 (PLK1); apoptosis inducing factor (AIF); Bcl-2-associated X protein (BAX); B-cell lymphoma 2 (BCL2); signal transducer and activator of transcription 3 (STAT3); light chain 3 phosphatidylethanolamine conjugate (LC3-II); N-methyl-D-aspartate receptor (NMDAR); vascular-endothelial growth factor 2 (VEGF); cyclooxygenase 2 (COX2); mechanistic target of rapamycin (mTOR); myeloid cell leukemia-1 (MCL-1); tyrosine kinase (TK); phosphoinositide 3-kinase (PI3K); carbonic anhydrase (CA); B-cell lymphoma 3-encoded protein (BCL-3); programmed cell death 1 (PD-1); cytotoxic T-lymphocyte-associated protein 4 (CTLA4); AMP-activated protein kinase (AMPK); epidermal growth factor receptor (EGFR); glycogen synthase kinase (GSK) 3β; deoxyribonucleic acid (DNA).

## Data Availability

No new data were created or analyzed in this study.
